# Supplementary material for: Tuning the Molecular Structure of Corroles to Enhance the Antibacterial Photosensitizing Activity
Source: Pharmaceutics. 2023 Jan 24;15(2):392. doi: 10.3390/pharmaceutics15020392 (PMC9959985; doi:10.3390/pharmaceutics15020392)
Supplement: Supplementary file 1 [file pharmaceutics-15-00392-s001.zip › pharmaceutics-2118833-supplementary.pdf]

Tuning the molecular structure of corroles to enhance the antibacterial photosensitizing activity

Edwin J. Gonzalez Lopez [1,†], Sol R. Martinez [2,†], Virginia Aiassa [3], Sofía C. Santamarina [1], Rodrigo E. Dominguez [4], Edgardo N. Durantini [1] and Daniel A. Heredia [1,*]

1- IDAS-CONCIET, Departamento de Química, Facultad de Ciencias Exactas Físico-Químicas y Naturales, Universidad Nacional de Río Cuarto, Agencia Postal Nro. 3, X5804BYA Río Cuarto, Córdoba, Argentina.
2- IITEMA-CONICET, Departamento de Química, Facultad de Ciencias Exactas Físico-Químicas y Naturales, Universidad Nacional de Río Cuarto, Agencia Postal Nro. 3, X5804BYA Río Cuarto, Córdoba, Argentina.
3- UNITEFA-CONICET, Departamento de Ciencias Farmacéuticas, Facultad de Ciencias Químicas, Universidad Nacional de Córdoba, X5000HUA Córdoba, Argentina.
4- INFIQC-CONICET, Departamento de Química Orgánica, Facultad de Ciencias Químicas, Universidad Nacional de Córdoba, X5000HUA Córdoba, Argentina.
† These authors contributed equally to this work.

*****Correspondence: dheredia@exa.unrc.edu.ar; Tel.: +54 (0358) 4676538.

Table of Contents

Description	Page Nº
**Experimental Procedures. Materials and Instrumentation**	S-3
**Figure S1.** Stability of the corrole derivatives in PBS.	S-5
**Figure S2**. ^{1}H and ^{19}F NMR spectra of compound **Co** in $CDCl_3$.	S-6
**Figure S3**. ^{1}H and ^{19}F NMR spectra of compound **Co-CF$_3$** in $CDCl_3$.	S-7
**Figure S4**. COSY NMR spectrum of compound **Co-CF$_3$** in $CDCl_3$.	S-8
**Figure S5**. ^{1}H and ^{19}F NMR spectra of compound **Co-CF$_3$-2NMe$_2$** in $CDCl_3$.	S-9
**Figure S6**. COSY and ^{13}C NMR spectra of compound **Co-CF$_3$-2NMe$_2$** in $CDCl_3$.	S-10
**Figure S7**. HSQC NMR spectrum of compound **Co-CF$_3$-2NMe$_2$** in $CDCl_3$.	S-11

Figure S8. ^{1}H and ^{19}F NMR spectra of compound **Co-3NMe$_2$** in DMSO-d$_6$. S-12

Figure S9. COSY and ^{13}C NMR spectra of compound **Co-3NMe$_2$** in DMSO-d$_6$. S-13

Figure S10. HSQC NMR spectrum of compound **Co-3NMe$_2$** in DMSO-d$_6$. S-14

Figure S11. Normalized fluorescence excitation spectra of corrole derivatives. S-15

Figure S12. Absorption spectra changes for DMA photooxidation. S-16

Figure S13. Drop in Log10 in CFU/mL profile of MRSA and K. pneumoniae upon light exposure. S-17

Figure S14. Dark controls of PDI treatments. S-17

Scheme S1. Type I and type II photodynamic mechanisms. S-18

Scheme S2. Photodecomposition of DMA mediated by $^1O_2$. S-18

Scheme S3. Reduction of NBT mediated by $O_2^{\bullet-}$. S-18

Equation S1. Fluorescence quantum yield. S-19

Equation S2. Quantum yield of $^1O_2$ production. S-20

Image S1. TLC photo of the nucleophilic aromatic substitution reaction using **Co** and 2-(dimethylamino)ethylamine. S-21

Experimental Procedures. Materials and Instrumentation

All chemicals were commercially acquired from Sigma-Aldrich and used without further purification. Solvents were stored with 3Å molecular sieves for at least 24 h before use. Molecular sieves were pre-dried at 300 °C for 24 h immediately before use. The reactions were monitored by TLC (silica gel 60 GF254) using different solvent mixtures. Flash column chromatographies were performed in silica gel 60 H (0,040-0,063 mM, 230-400 mesh ASTM, Merck) by gradient elution of mixture of petroleum ether/DCM or DCM/MeOH, under positive pressure of nitrogen. Silica gel 60 (0.040–0.063 mm, 230-400 mesh) from Merck was used for flash column chromatography. Tryptic soy broth, Sabouraud glucose broth and agar from Britania (Buenos Aires, Argentina) were used in microbial cultures. Microtiter plates (96-well) were acquired from Deltalab (Barcelona, Spain).

Nuclear magnetic resonance (NMR) spectra were performed on a FT-NMR Bruker Advance DPX400 at 400 MHz (Bruker BioSpin, Rheinstetten, Germany). The NMR spectroscopic data were recorded in $CDCl_3$ and DMSO-d_6 for Co-3NMe_2. Chemical shifts are reported in parts per million on the δ scale, and Me_4Si was used as the internal standard. Two-dimensional NMR experiments (2D NMR) (COSY, homonuclear correlation spectroscopy, HSQC, heteronuclear single quantum correlation spectroscopy) were also employed. The magnitude of the coupling constants (*J*) is given in Hertz. Mass spectra were recorded on a Bruker micrOTOF-QII (Bruker Daltonics, MA, USA) equipped with an ESI source (ESI-MS).

Absorption spectra were carried out on a Shimadzu UV-2401PC spectrometer (Shimadzu Corporation, Tokyo, Japan). Fluorescence spectra were performed on FluoroMax-4 spectrofluorometer (Horiba Jobin Yvon Inc, Edison, NJ, USA). Spectroscopic and photodynamic determinations were performed in a quartz cell of 1 cm

path length at room temperature. Cell growth was measured with a Turner SP-830 spectrophotometer (Dubuque, IA, USA). Fluence rates were obtained with a Radiometer Laser Mate-Q (Coherent, Santa Clara, CA, USA). Photolysis of DMA and NBT experiments were performed with a Cole-Parmer illuminator 41720-series (150 W halogen lamp, Cole-Parmer, Vernon Hills, IL, USA) in combination with a high intensity grating monochromator (Photon Technology Instrument, Birmingham, NJ, USA). This arrangement produces a light fluence rate of 1.15 mW cm^{-2} at 606 nm ± 5 nm and 1.18 mW cm^{-2} at 628 nm ± 5 nm. The experiments of DMA and NBT were repeated in three independent measurements. Results represent means ± standard deviation of the three independent experiments.

Cell suspensions were irradiated with a Novamat 130 AF (Braun Photo Technik, Nürnberg, Germany) slide projector containing a 150 W 24 V halogen lamp (Osram, Munich, Germany). A 2.5 cm glass cuvette filled with water without circulation was used to remove the heat from the lamp. For visible light irradiation a wavelength range between 350 and 800 nm was selected by optical filters. The projector was placed vertically with the light beam focused on the 96-well microtiter plate lid, producing a fluence rate of 90 mW/cm^2.

Controls and Statistical Analysis.

The experimental values represent the mean obtained from three independent determinations under the same conditions and the error bars denote the standard deviation. Controls of bacteria were attained with irradiated cultures without corrole and in presence of PS in the dark. The unpaired *t*-test was used to establish the significance of differences between groups. Differences between means were tested for significance by one-way ANOVA. Results were statistically significant using a confidence level of 95% ($p<0.05$).

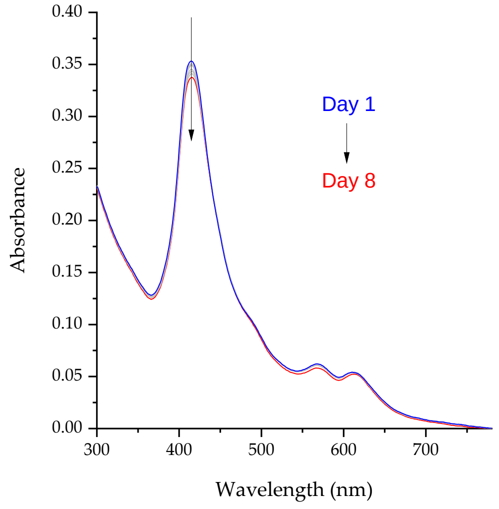

Figure S1. Spectral changes of the corrole unit (Co-3NMe$_2$) after incubation in 2 mL of PBS in a closed quartz cuvette kept in the dark at room temperature.

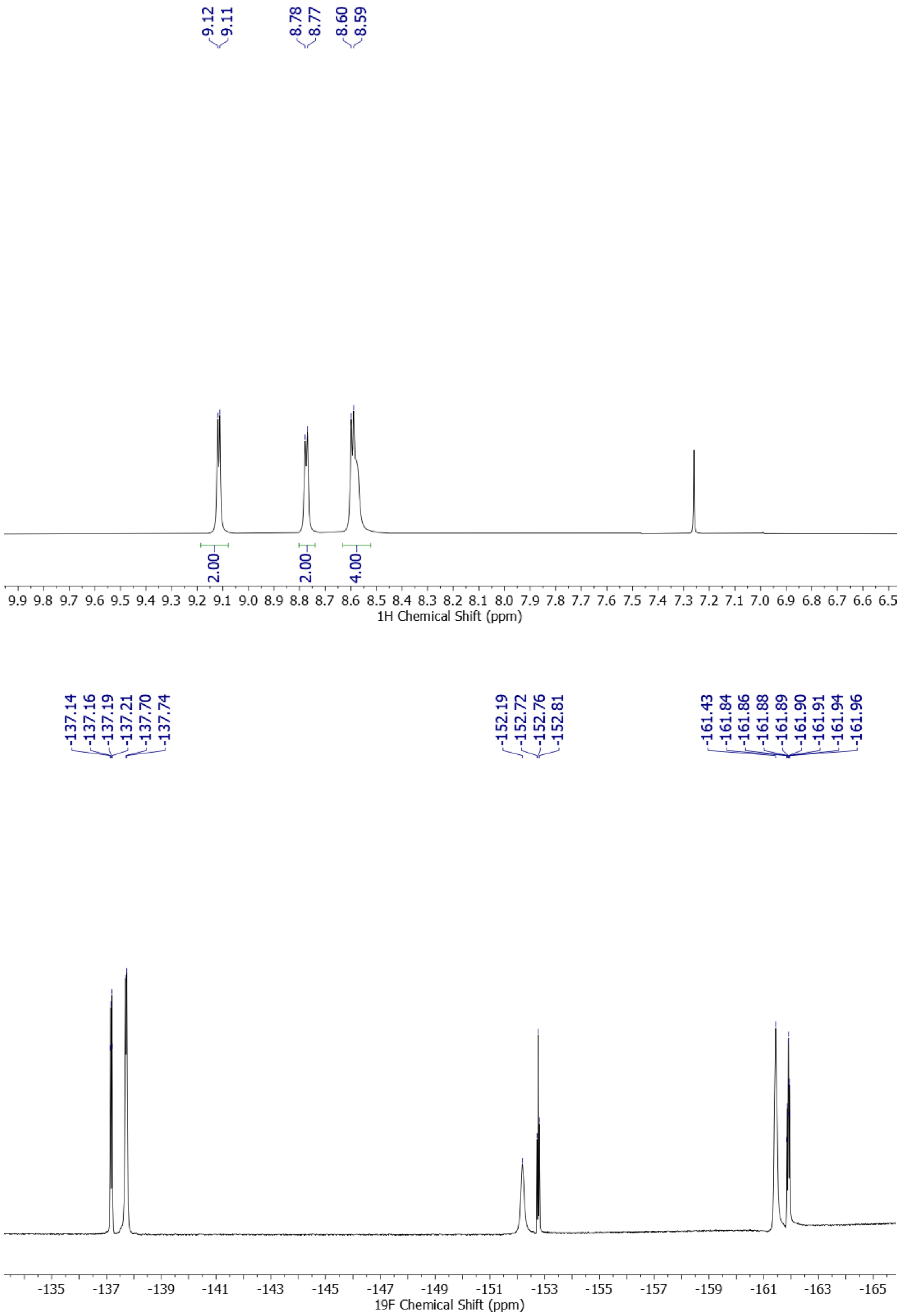

Figure S2. ^{1}H and ^{19}F NMR spectra of compound **Co** in $CDCl_3$.

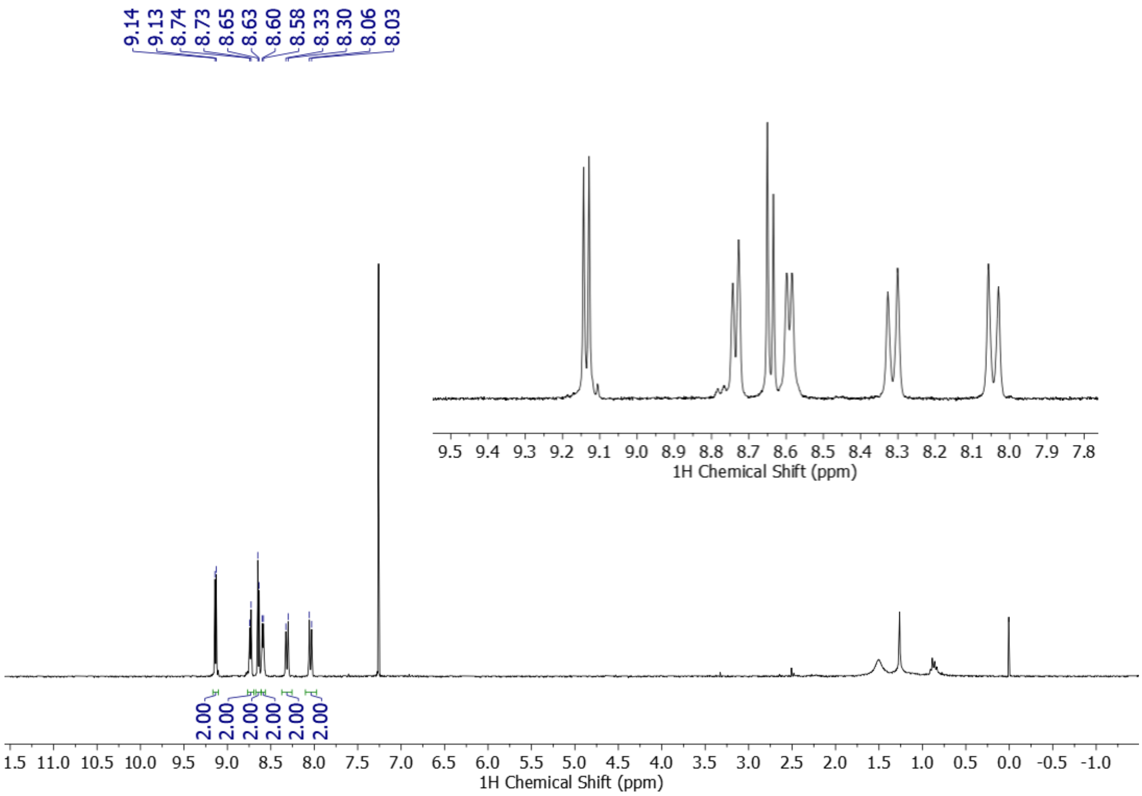

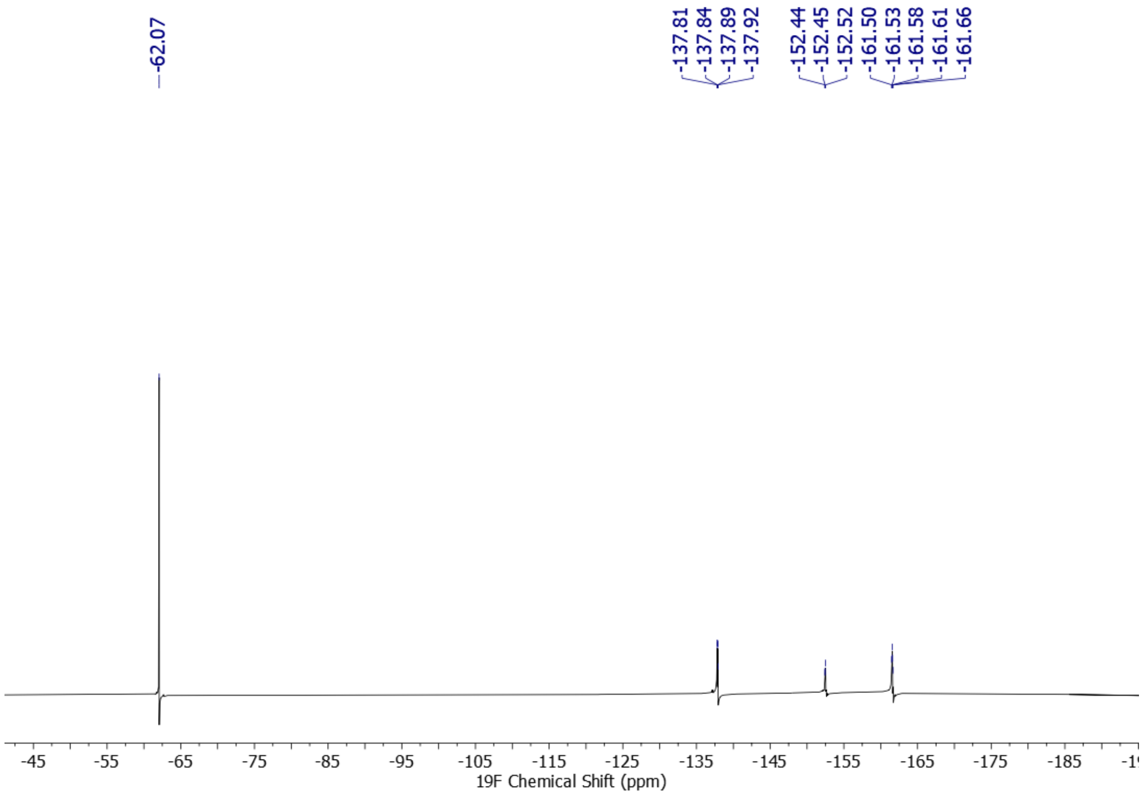

Figure S3. ${}^{1}H$ and ${}^{19}F$ NMR spectra of compound **Co-CF$_3$** in $CDCl_3$.

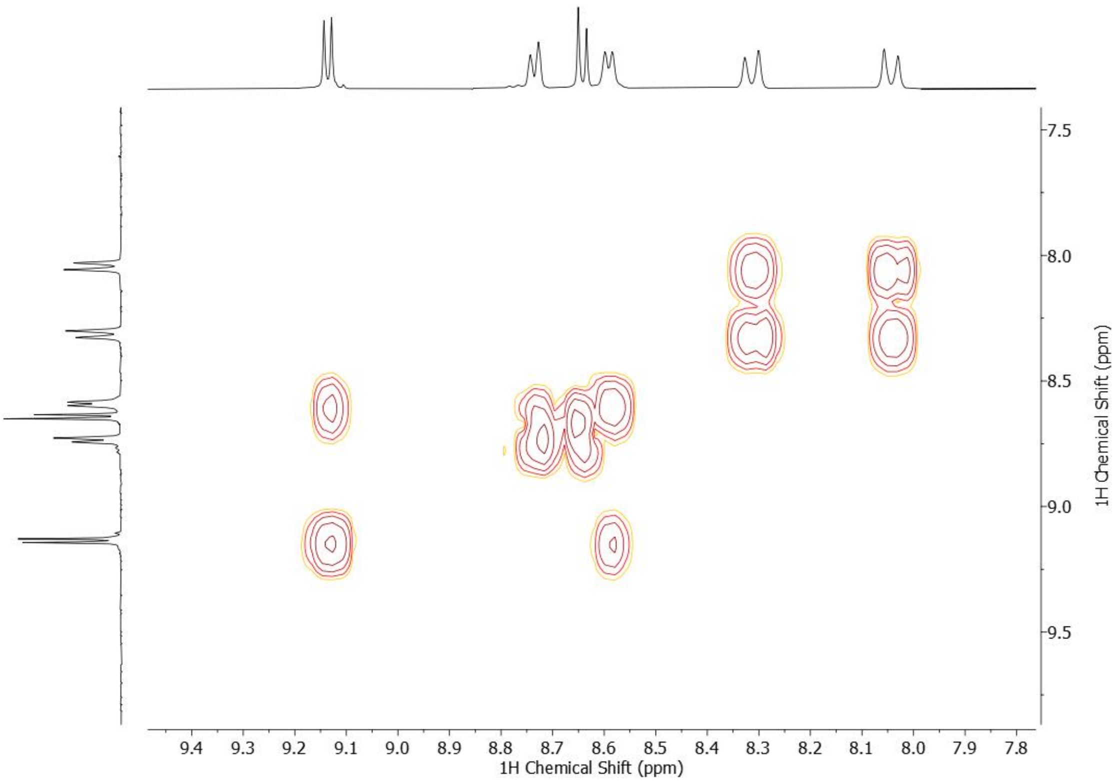

Figure S4. COSY NMR spectrum of compound **Co-CF$_3$** in $CDCl_3$

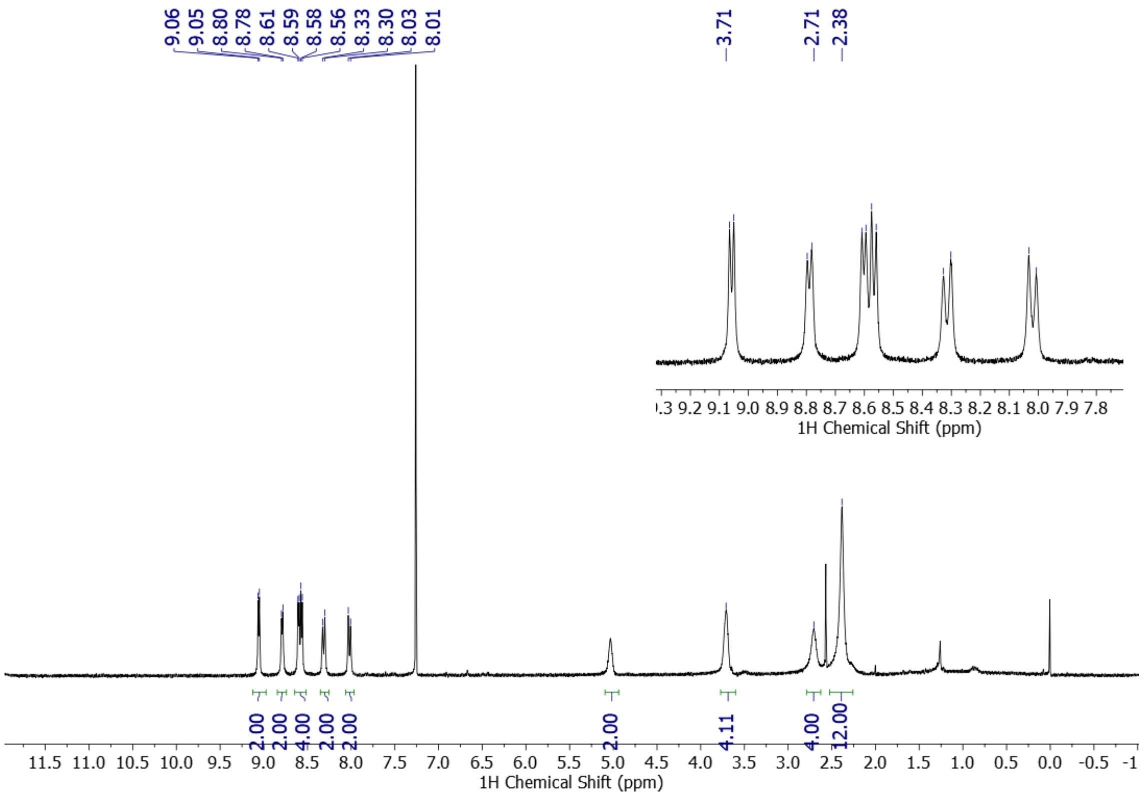

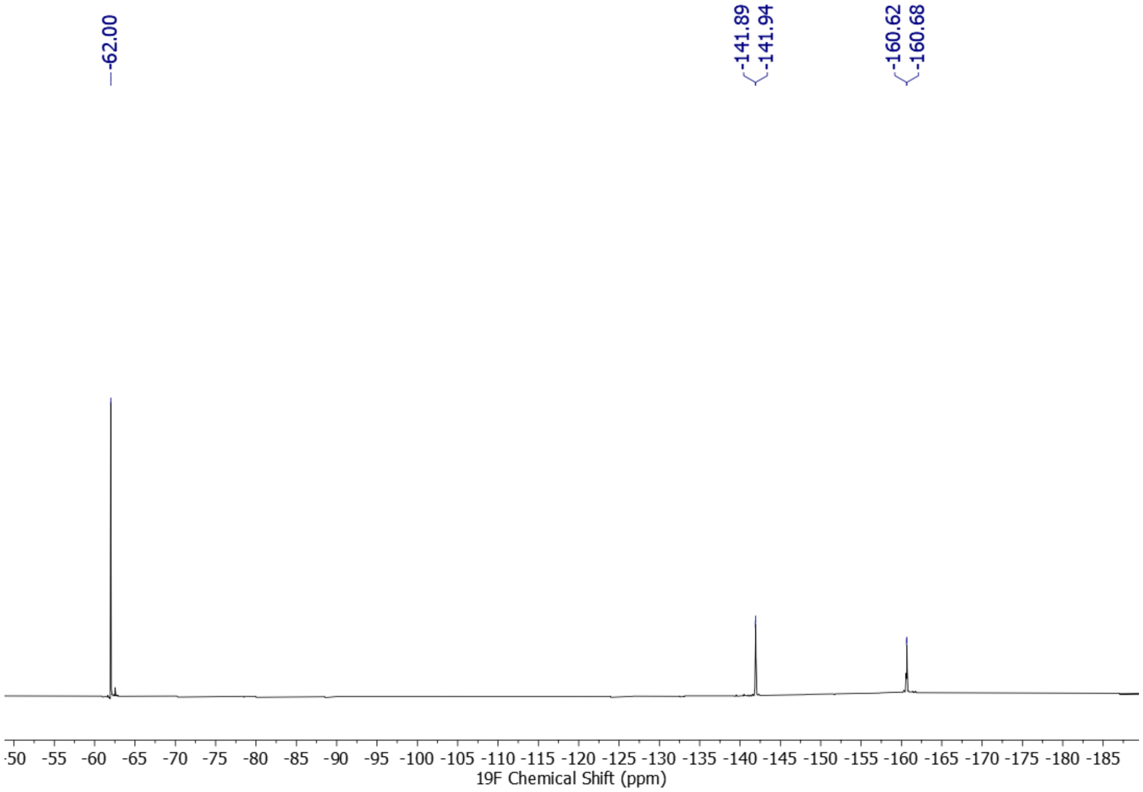

Figure S5. ^{1}H and ^{19}F NMR spectra of compound **Co-CF$_3$-2NMe$_2$** in $CDCl_3$.

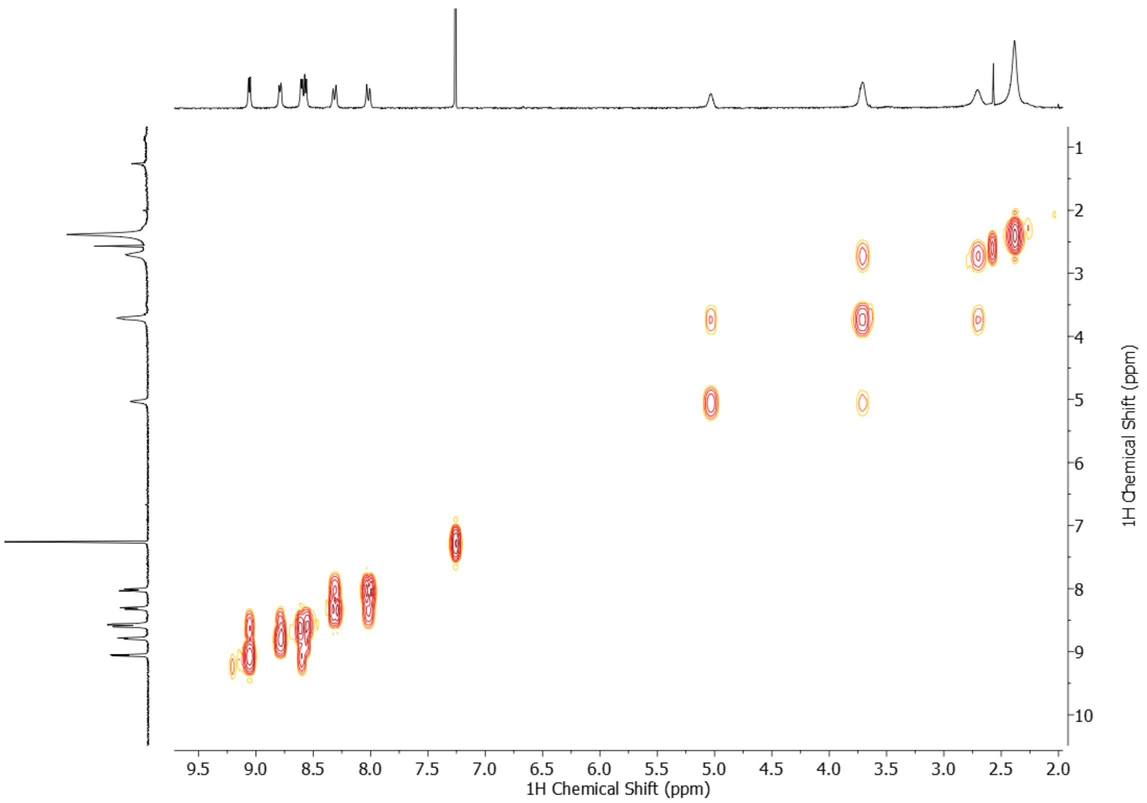

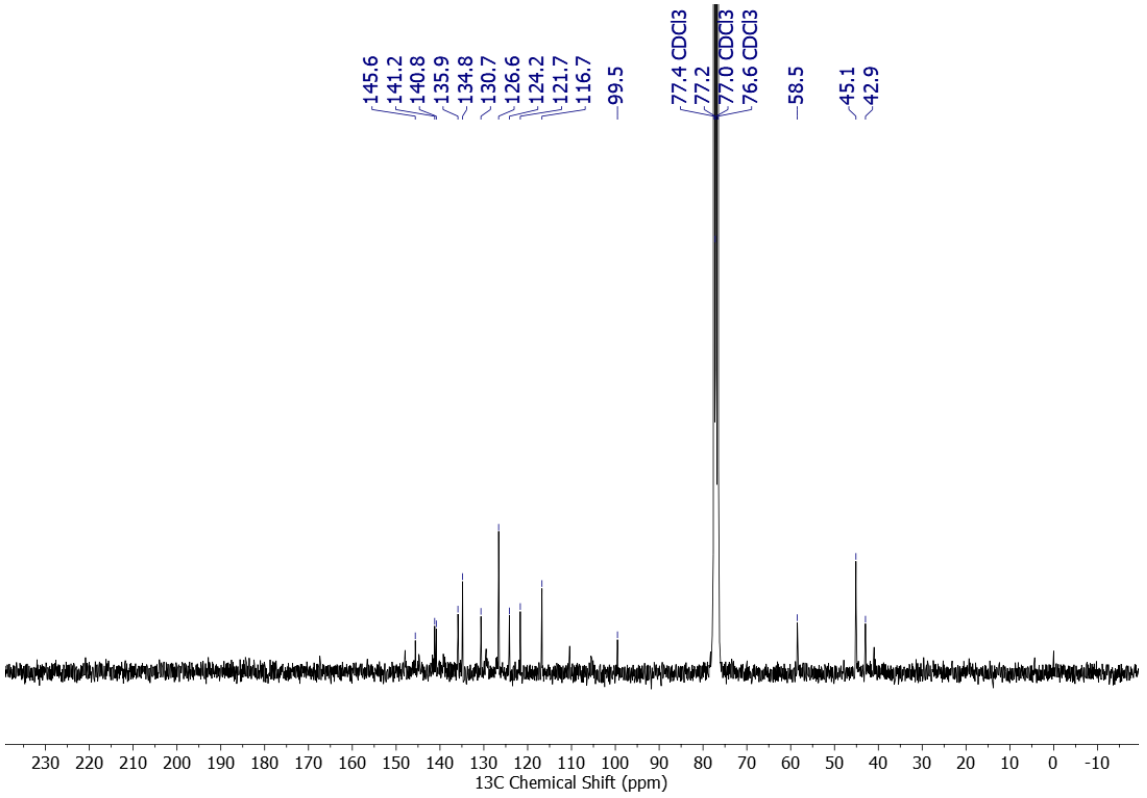

Figure S6. COSY and ^{13}C NMR spectra of compound **Co-CF$_3$-2NMe$_2$** in $CDCl_3$.

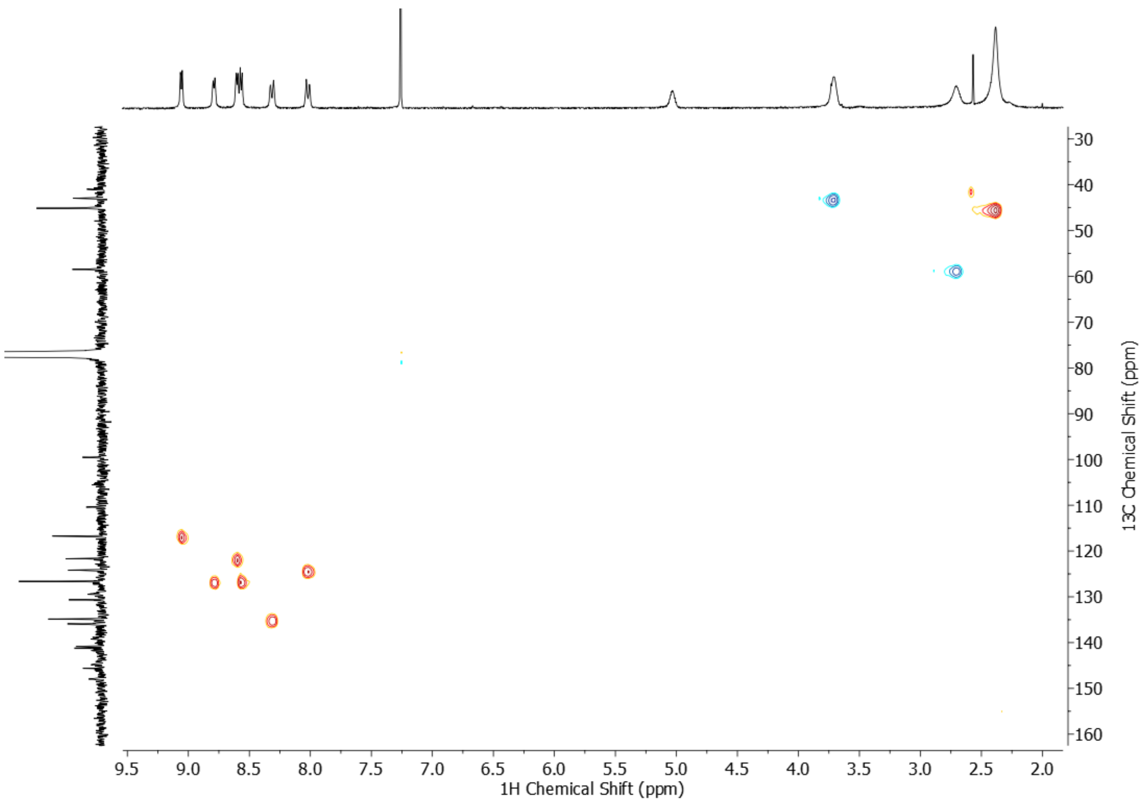

Figure S7. HSQC NMR spectrum of compound **Co-CF3-2NMe2** in $CDCl_3$.

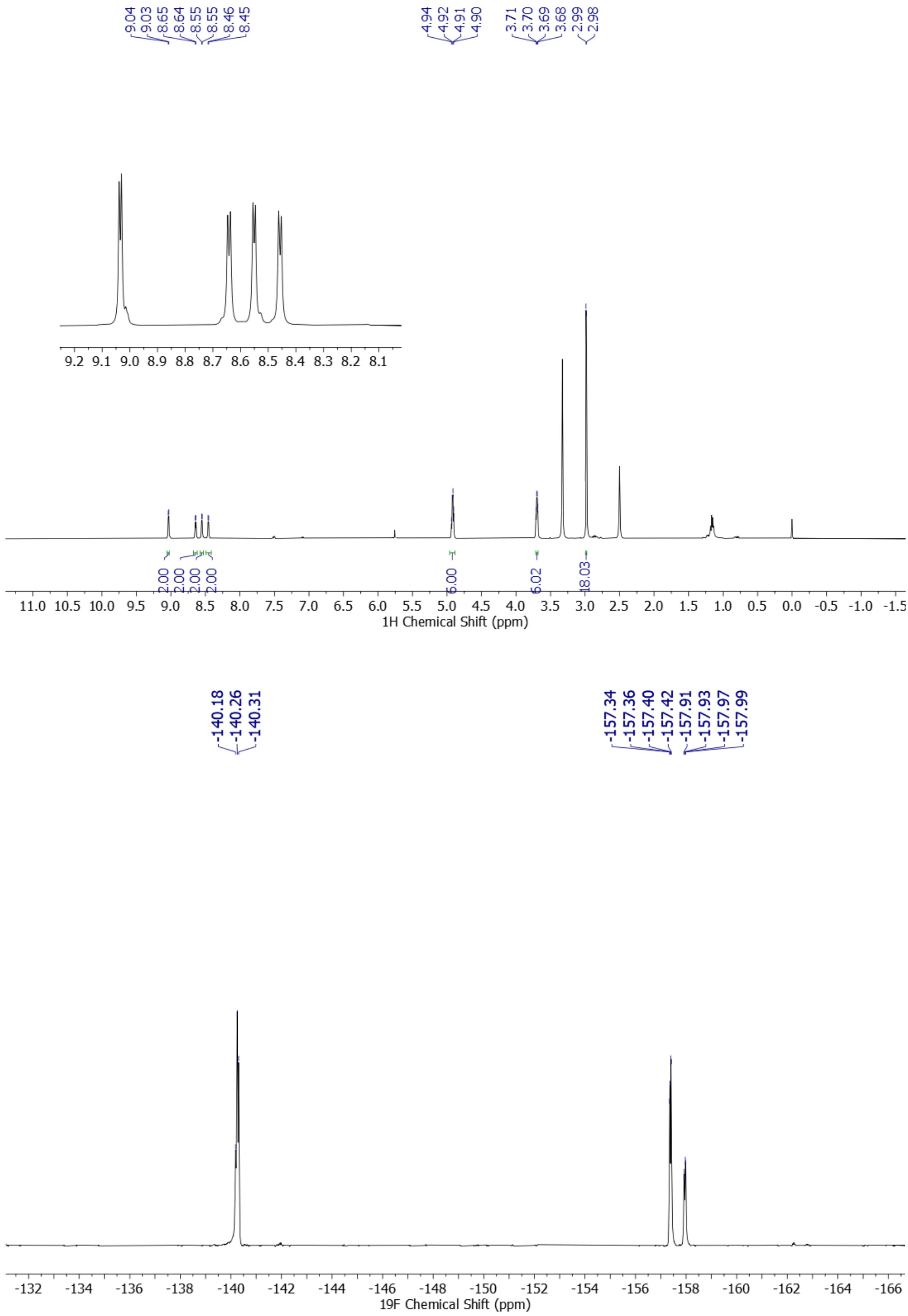

Figure S8. ^{1}H and ^{19}F NMR spectra of compound **Co-3NMe$_2$** in DMSO-d$_6$.

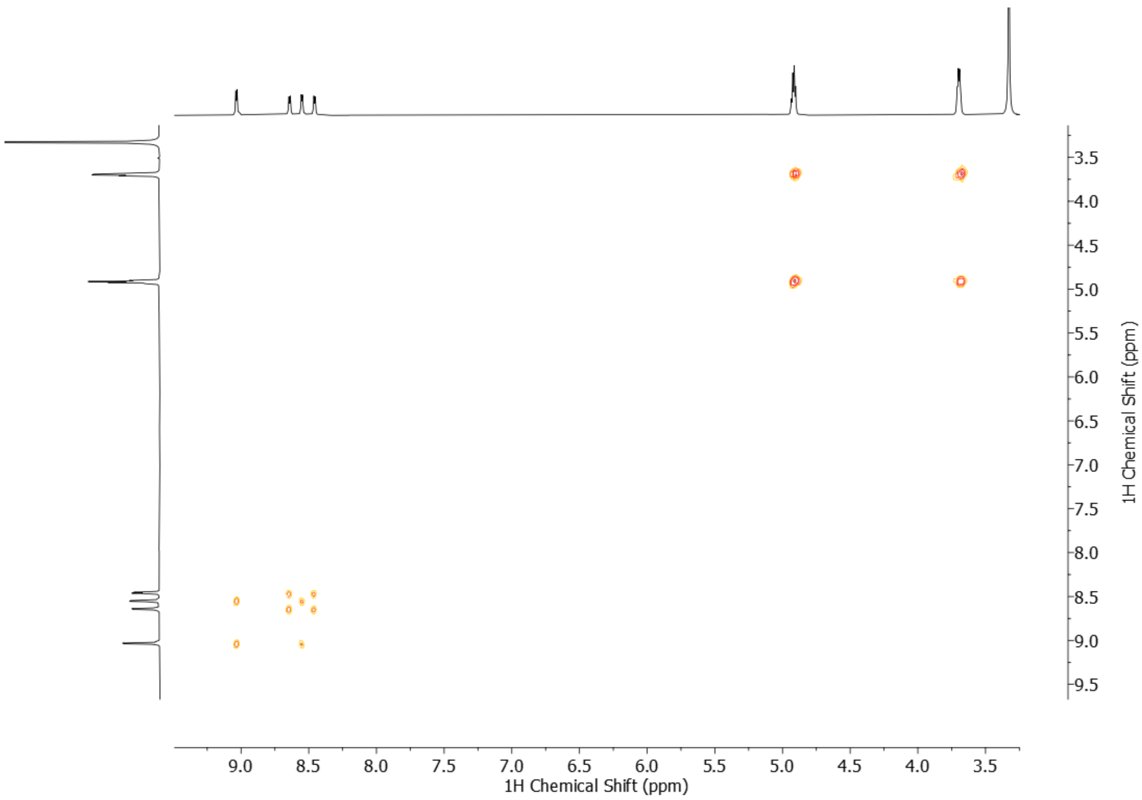

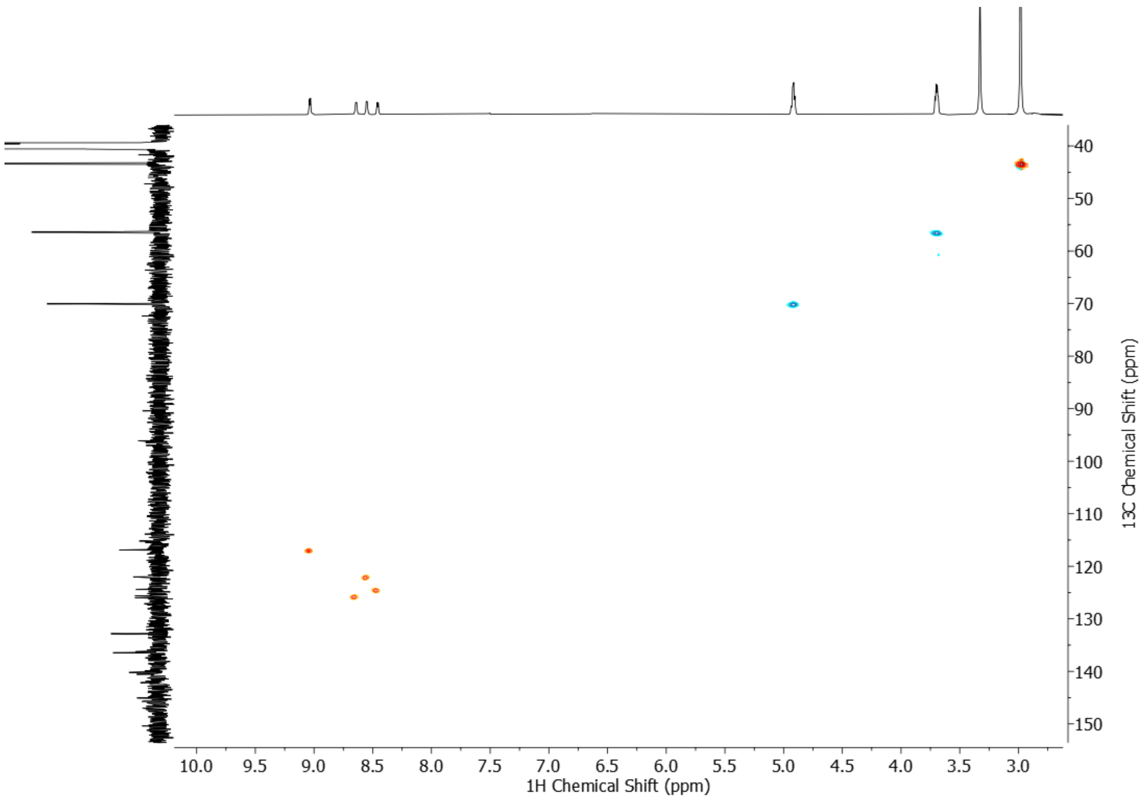

Figure S9. COSY and ^{13}C NMR spectra of compound **Co-3NMe$_2$** in DMSO- d_6.

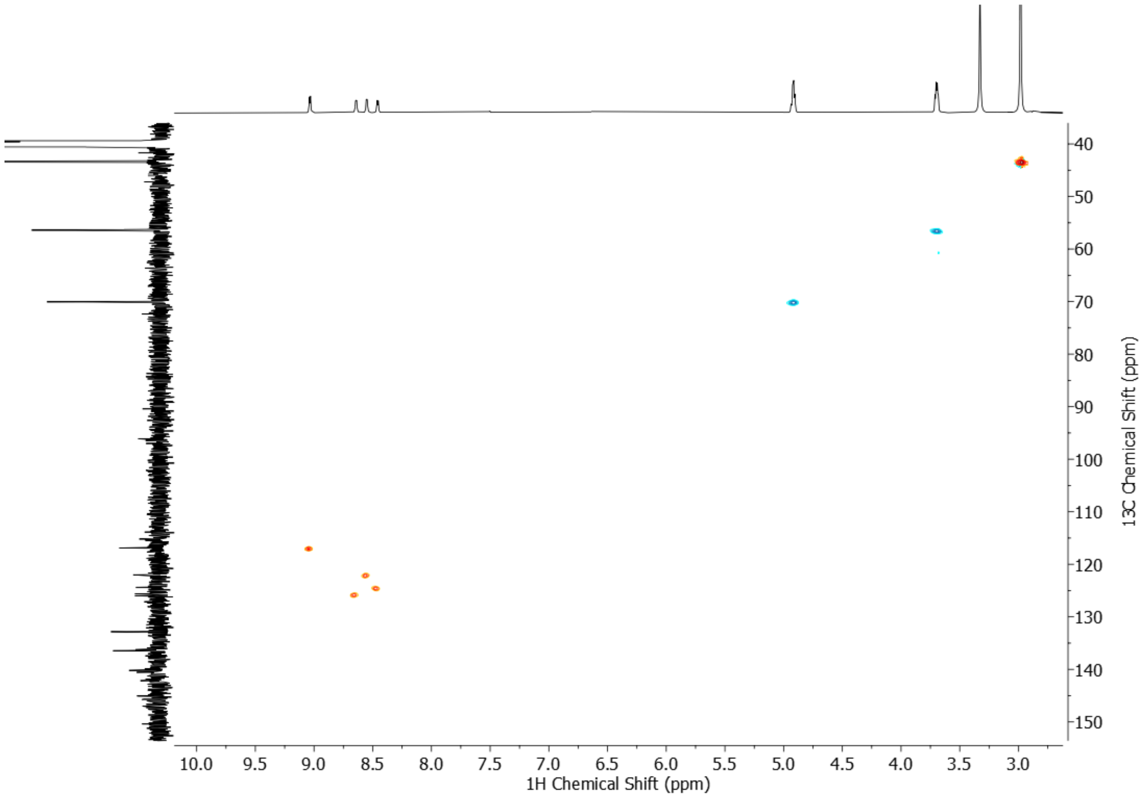

\

Figure S10. HSQC NMR spectrum of compound **Co-3NMe$_2$** in DMSO-d$_6$.

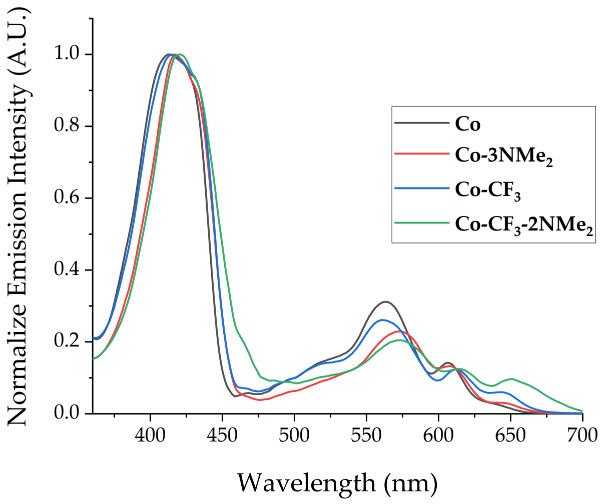

Figure S11. Normalized fluorescence excitation spectra of **Co** (black), **Co-CF$_3$** (blue) **Co-3NMe$_2$** (red) and **Co-CF$_3$-2NMe$_2$** (green) in toluene (λ_{em} = 710 nm).

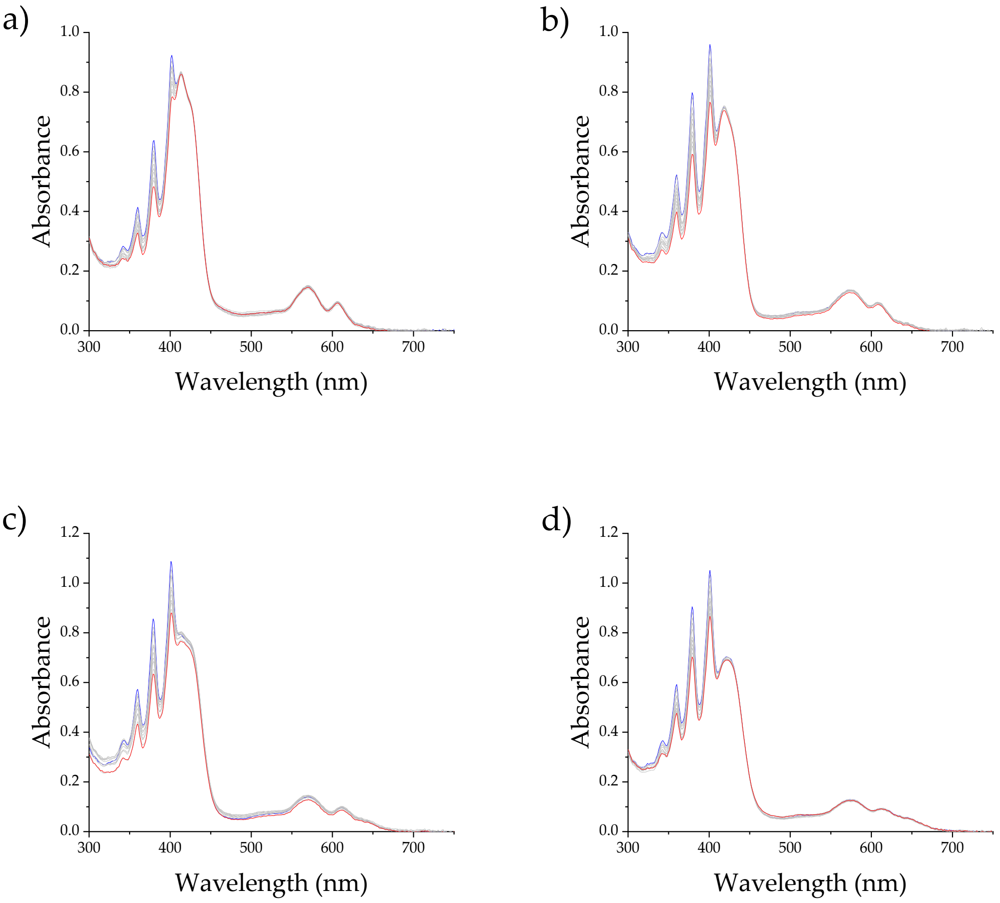

Figure S12. Absorption spectra changes for DMA photooxidation in toluene photosensitized by (a) **Co**, b) **Co-3NMe$_2$**, c) **Co-CF$_3$**, and d) **Co-CF$_3$-2NMe$_2$**, after different irradiation times. λ_{irr} = 606 nm nm.

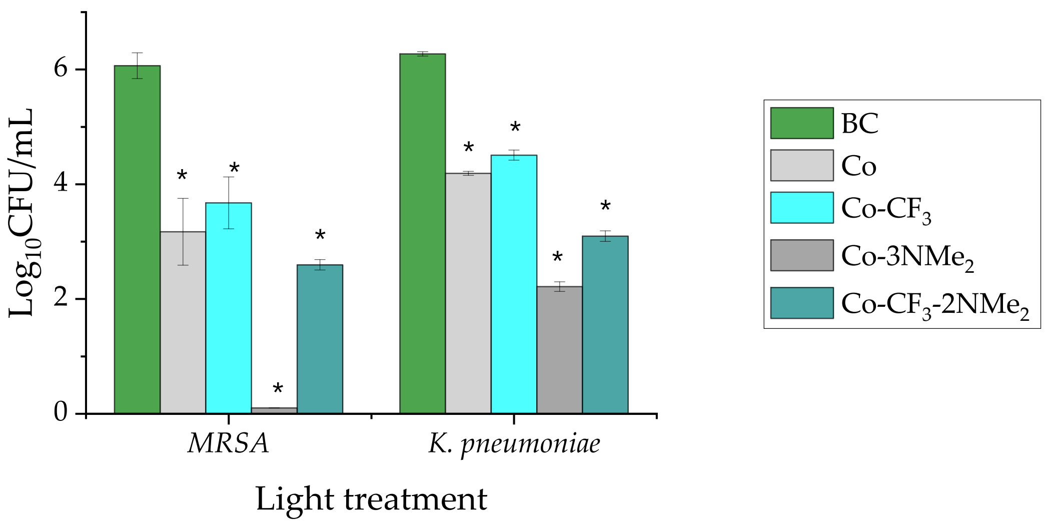

Figure S13. Drop in Log10 in CFU/mL profile of MRSA and *K. pneumoniae* upon light exposure. The following colors represent each compound: dark gray, **Co-3NMe2**; dark cyan, **Co-CF3-2NMe2**; light gray **Co** and light cyan **Co-CF3**. Green bar indicates the bacteria control exposed to light without any compound. Results are represented as the mean ± standard deviation of 3 replicates performed in three independent days.

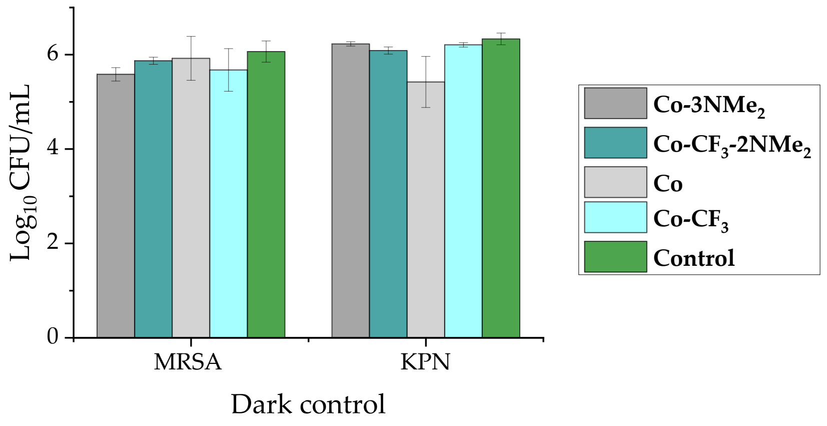

Figure S14. Cell survival profile of MRSA and *K. pneumoniae* treated with **Co-3NMe2** (dark gray); **Co-CF3-2NMe2** (dark cyan), **Co** (light gray) and **Co-CF3** (light cyan) for 20 min at 37 ºC in the dark and kept in the dark for 30 min.

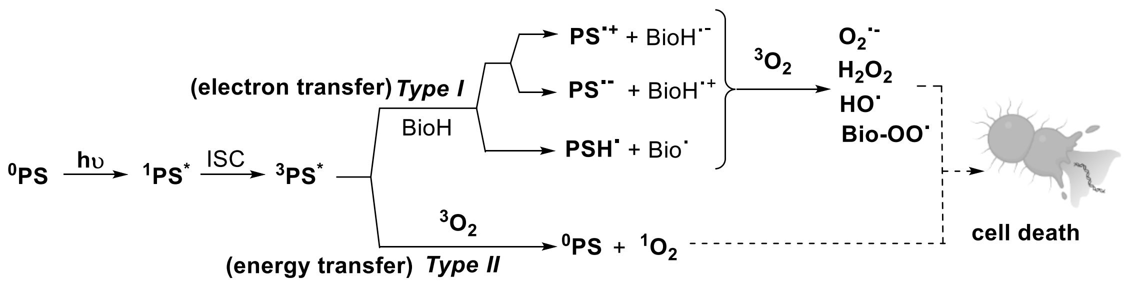

Scheme S1. Type I and type II photodynamic mechanisms.

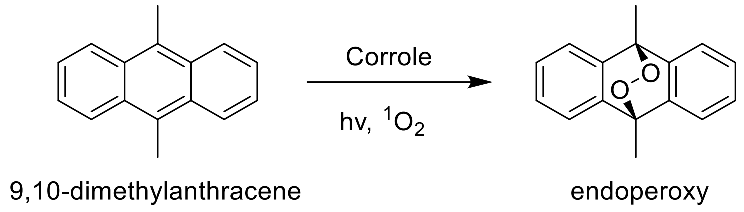

Scheme S2. Photodecomposition of DMA mediated by $^1O_2$ to produce 9,10-endoperoxide.

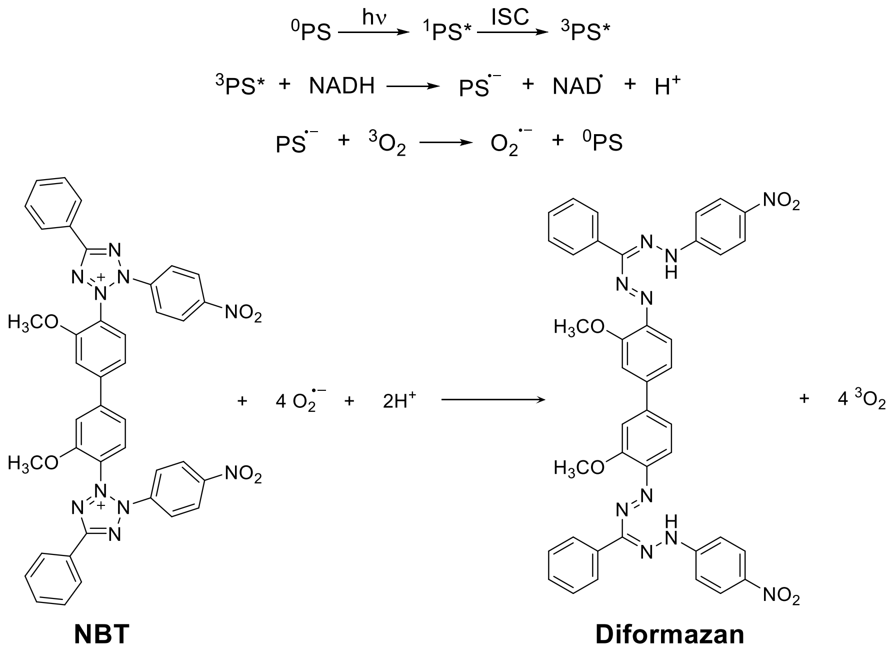

Scheme S3. Reduction of NBT mediated by $O_2^{\bullet-}$ to produce diformazan.

Fluorescence quantum yield (Φ) was calculated from eq. S1, comparing the areas under the emission spectra of the dye with the reference (**BP**, Φ = 0.92) in the 450-800 nm range.

$$\Phi^{Ps} = \frac{A^{Ref}}{A^{PS}} \times \frac{I^{PS}}{I^{Ref}} \times \left(\frac{\eta_{PS}}{\eta_{Ref}}\right)^2 \times \Phi^{Ref}$$

Equation S1.

In eq. S1, A is the absorbance (A < 0.07) at the excitation wavelength and I is the area of the emission spectra of the reference (Ref) and the photosensitizer (PS) under study. η is the refractive index the solvent.

Quantum yields of singlet oxygen production (Φ_Δ) were calculated by comparison of the observed rate constant ($k_{obs} = k_q [^1O_2]$) with that obtained for the reference. The sample and the references were measured under the same conditions. The k_{obs} were determined from the slopes of the semilogarithmic plot ln (A_0/A) *vs.* time (s). For the reaction of DPBF with $^1O_2$ in steady state, the disappearance rate of the substrate is:

$$\frac{\delta[DPBF]}{\delta t} = -k_q[1O_2][DPBF] = -k_{obs}[DPBF]$$

$$\int_{[DMA]_0}^{[DMA]_t} \frac{\partial[DPBF]}{[DPBF]} = -k_{obs}\int_o^t \partial t$$

$$ln(\frac{[DPBF]_t}{[DPBF]_0}) = -k_{obs} \cdot t$$

$$ln(\frac{[DPBF]_0}{[DPBF]_t}) = k_{obs} \cdot t$$

$$ln\frac{[DPBF]_0}{[DPBF]_t} = k_{obs}t$$

Replacing the DBPF concentrations by the corresponding absorbances at 414 nm:

$$A = \epsilon.b.[DPBF]$$

$$ln\frac{[A]_0}{[A]_t} = k_{obs}t$$

The Φ_Δ for each PS was calculated by direct comparison of the slopes in the linear region of the plot between the reference and the PS (for the same absorbance of the Ref and PS at the irradiation wavelength):

$$\Phi_\Delta^{PS} = \frac{\Phi_\Delta^{Ref}\, k_{obs}^{PS}}{k_{obs}^{Ref}}$$

Equation S2.

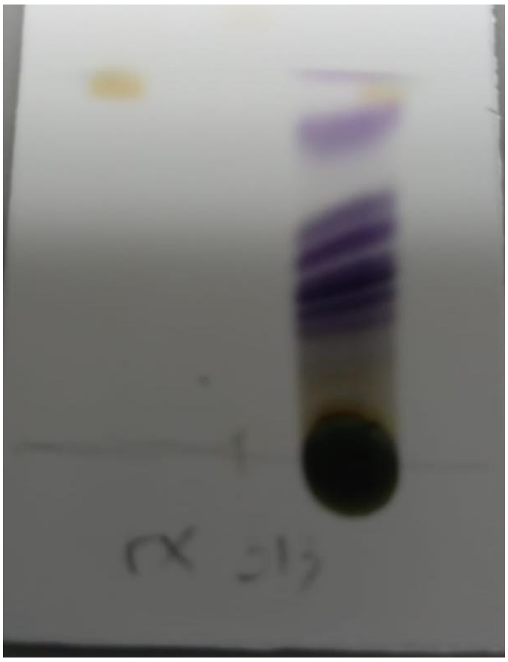

Image S1. TLC photo of the nucleophilic aromatic substitution reaction using **Co** and 2-(dimethylamino)ethylamine.
